# Cancer survival in Malawi: a retrospective cohort study

**DOI:** 10.11604/pamj.2014.19.234.4675

**Published:** 2014-10-31

**Authors:** Kelias Phiri Msyamboza, Geoffrey Manda, Bvumi Tembo, Chimwemwe Thambo, Linly Chitete, Christopher Mindiera, Lucy Kishindo Finch, Kathryn Hamling

**Affiliations:** 1 World Health Organisation, Malawi Country Office, Lilongwe, Malawi; 2 University of Malawi, College of Medicine, Community Health Department, Lilongwe, Malawi; 3 Ndimoyo Palliative Care Centre, Salima, Malawi

**Keywords:** Cancer, cancer survival, palliative care, sub-Saharan Africa, Malawi

## Abstract

**Introduction:**

Cancer is a leading cause of morbidity and mortality worldwide with the burden in sub-Saharan Africa projected to double by year 2030 from 715,000 new cases and 542,000 deaths in 2008. However, cancer survival data to inform interventions for early detection, diagnosis and treatment are lacking.

**Methods:**

Cancer survival analysis was conducted on 842 cancer patients registered and followed-up from 2006 to 2013 at NdiMoyo Palliative Care Centre in Salima District, central Malawi. Cancer survival was measured from the time of diagnosis.

**Results:**

In both sexes, the common types of cancer were; Kaposi's sarcoma (KS) (48.0%), cervical cancer (21.1%), cancer of oesophagus (14.8%), liver cancer (3.1%) and breast cancer (2.5%). In Males; KS, cancer of the oesophagus, cancer of the liver, bone cancer and non-Hodgkin's lymphoma were the commonest accounting for 67.4%, 19.4%, 3.9%, 1.0% and 1.0% respectively. In females; cancer of the cervix, KS, cancer of the oesophagus, cancer of the breast and cancer of the liver were the top five cancers accounting for 41.6%, 29.2%, 10.3%, 4.9% and 2.3% respectively. Of the 830 cancer patients with complete 5-year follow-up data, the overall median survival time was 9 months. Absolute survival rates at 1, 2, 3, 4 and 5 years or more were 31.8%, 18.0%, 12.5%, 7.8% and 6.0% respectively. The survival rates for top five cancers at 1, 2, 3, and 4 years or more were; KS (n= 397): 47.1%, 30.2%, 21.4% and 13.1%; cancer of the cervix (n = 174): 31.0%, 10.3%, 5.2% and 2.9%; cancer of the oesophagus (n = 124): 4.0%, 2.4%, 1.6% and 1.6%; liver cancer (n = 26): 19.2%, 3.8%, 3.8% and 3.8% and breast cancer (n = 21): 9.5%, 0%, 0%, 0% respectively. The risk of death was high in females than males, in those aged 50 years or more than in those aged less than 50 (p < 0.05).

**Conclusion:**

This study demonstrated that cancer survival from the time of diagnosis in Malawi was poor with median survival time of about 9 months and only 6% of patients survived for 5 years or more. Improvement of early detection, diagnostic capability, access to treatment and palliative care services could improve cancer survival.

## Introduction

Cancer is a leading cause of morbidity and mortality worldwide. In 2008, globally, there were 12.7 million new cancer cases and 7.6 million cancer deaths (around 13% of all deaths) with 56% of the new cases and 63% of the cancer deaths occurring in developing countries. It is projected that by 2030, the number of new cancer cases and deaths will increase by 69% and 72% to 21.4 million and 13.2 million respectively [1–3].

In sub-Saharan Africa, it has been projected that the burden of cancer will double by year 2030 from 715,000 new cases and 542,000 deaths in 2008 [3]. Cancer survival tends to be poor in this region because of a combination of a late stage at diagnosis and limited access to timely and standard treatment [4–6]. For example, in Uganda and Zimbabwe, 5-year relative survival for colorectal cancer and cervical cancer were as low as 8.3% and 17.7%, 17.4% and 30.5% compared with 63.9%, 58.1% respectively, for black American patients [7, 8]. At the time of diagnosis, over 80% of cancer patients are in advanced and incurable stage, making the need for palliative care more important in this region [9–11]. World Health Organization defines palliative care as an approach that improves the quality of life of patients and their families facing the problem associated with life-threatening illness, through the prevention and relief of suffering by means of early identification, assessment and treatment of physical, psychosocial and spiritual problems. Palliative care, if initiated soon after diagnosis, has been found to improve the treatment outcomes and survival of cancer patients [12, 13].

In Malawi, cancer is a major public health problem with estimated age-standardised incidence rate (ASR) per 100,000 population per year of 55.5 in males and 68.8 in females for all types of cancer. In females, cancer of the cervix is the commonest accounting for 45.4% of all cases followed by Kaposi's sarcoma (21.1%), cancer of the oesophagus (8.2%), breast (4.6%) and non-Hodgkin lymphoma (4.1%). In males, Kaposi's sarcoma is the commonest (50.7%) followed by cancer of oesophagus (16.9%), non-Hodgkin lymphoma (7.8%), prostate (4.0%) and urinary bladder (3.7%). In both sexes, the top five common cancers are; Kaposi's sarcoma (34.1%), cancer of the cervix (25.4%), oesophagus (12.0%), non-Hodgkin lymphoma (5.7%) and urinary bladder (2.9%) [14]. Comprehensive data on cancer survival to inform policies, strategies and interventions are scarce in most countries in eastern and southern Africa. In October and November 2013, a retrospective cohort study was conducted at NdiMoyo Palliative Care Centre, Salima, central Malawi to determine cancer survival rates.

## Methods

### Study design, place and data collection

This was retrospective cohort study of patients registered at NdiMoyo Palliative Care Centre in Salima district, central Malawi. NdiMoyo Palliative Care Centre was established in August 2006 as a stand-alone, non-governmental, day-care palliative centre. It is registered as a trust and it has 17 full time staff, 5 of whom are clinicians and the rest are support staff. In addition, it has 4 volunteers. It works closely with Salima District Hospital (a government main hospital in the district) where patients from NdiMoyo needing vincristine, diagnostic services or hospital admission are referred to. Clinicians from NdiMoyo conduct ward rounds at Salima District Hospital twice a week to help the government regardless whether there is a patient from NdiMoyo or not. Salima District Hospital and other health care facilities refer cancer patients, as soon as the diagnosis is made, to NdiMoyo Palliative Care Centre for comprehensive holistic palliative care. Patients of all ages and both sexes with single primary cancer registered between August 2006 and November 2013 were eligible for this analysis. Data were abstracted from registers and patient files using a standard data collection form. Cancer survival was measured from the time of diagnosis.

### Data management

Data was entered in Microsoft excel^®^ and exported to SPSS version 20 for analysis. Absolute Kaplan Meier survival estimates were determined at 1, 2, 3,4 and 5 years or more. Survival rates were dis-aggregated by gender and age. Cox proportional hazards method was used to test for significance of age or sex on survival by calculating hazards ratio. Logrank test was used to test for difference in survival curves between different groups. Data was also summarized into descriptive statistics. All analyses were made at 95% confidence level and p < 0.05 was considered statistically significant.

### Ethics statement

Ethical approval was granted by the College of Medicine Ethics Committee (COMREC). Informed written consent to extract data from hospital registers and patients’ files was obtained from directors of NdiMoyo Palliative Care Centre.

## Results

### Characteristics of cancer cases enrolled in the study

A total of 860 cancer patients were registered at NdiMoyo Palliative Care Centre from 2006 to 2013. Out of these, 18 (2.1%) had incomplete follow-up data and were therefore excluded from this cancer survival analysis. Of the 842 eligible patients, 414 (49.2%) were males and 428 (50.8%) were females; 15 (1.8%) were children under 15 years old, 659 (78.2%) were aged 15-59 years and 168 (20.0%) were elderly aged 60 years or more; 475 (56.4%) were HIV positive. At the time of study (November 2013), of 842 cancer patients ever registered at NdiMoyo, 63.9% were dead, 30.2% were still alive and 5.9% were lost to follow-up after a minimum follow-up period of 3 months (Table 1).

**Table 1:** Characteristics of cancer cases enrolled in the cancer survival analysis: NdiMoyo Palliative Care Centre, Malawi: 2006-2013

	Total		Male		Female	
	n	%	n	%	n	%
**Sex**	842	100	414	49.2	428	50.8
**Age (years):**						
≤14	15	1.8	8	1.9	7	1.6
15-29	108	12.8	51	12.3	57	13.3
30-44	343	40.7	199	48.1	144	33.6
45-59	208	24.7	97	23.4	111	25.9
≥60	168	20.0	59	14.3	109	25.5
Total with known age	842	100	414	49.2	428	50.8
**Education:**						
None	229	28.2	75	18.9	154	37.6
Primary	448	55.2	236	59.5	212	51.6
Secondary	114	14.1	74	18.6	40	9.8
Tertiary	20	2.5	12	3.0	4	1.0
Total with known education level	811	100.0	397	100	410	100
**Marital status:**						
Married	562	68.8	325	80.6	237	57.3
Single	45	5.5	29	7.2	16	3.9
Divorced/separated	106	13.0	37	9.2	69	16.7
Widowed	104	12.7	12	3.0	92	22.1
Total with known marital status	817	100	403	100	414	100
**Occupation:**						
Employed	86	10.4	62	15.3	24	5.7
Self-employed	228	27.6	145	35.9	83	19.7
Unemployed	509	61.7	195	48.3	314	74.6
Student	2	0.3	2	0.5	0	0
Total with known occupation status	825	100	404	100	421	100
**HIV status:**						
Negative	233	27.7	86	20.8	147	34.3
Positive	475	56.4	289	69.8	186	43.5
Unknown	134	15.9	39	9.4	95	22.2
Total	842	100	414	100	428	100

n= number in the group,%= percentage

### Common types of cancer registered at NdiMoyo Palliative Care Centre

Of the 842 cancer cases, 404 (48.0%) had Kaposi's sarcoma (KS), 178 (21.1%) had cervical cancer, 125 (14.8%) had cancer of oesophagus, 26 (3.1%) had liver cancer and 21 (2.5%) had breast cancer. In Males; KS, cancer of the oesophagus, cancer of the liver, bone cancer and non-Hodgkin's lymphoma were the commonest accounting for 67.4%, 19.4%, 3.9%, 1.0% and 1.0% respectively. In females; cancer of the cervix, KS, cancer of the oesophagus, cancer of the breast and cancer of the liver were the top five cancers accounting for 41.6%, 29.2%, 10.3%, 4.9% and 2.3% respectively. Of the 404, 178, 125, 26, and 21 KS, cervical cancer, cancer of the oesophagus, liver and breast cancer cases; 98.0%, 28.7%, 5.6%, 11.5% and 9.5% were HIV positive respectively. About 96.3% of cancer patients were clinically diagnosed whereas only 3.7% were histologically confirmed. None of the patients had clinical or histological stage of the cancer documented. Most of the cancer diagnoses (87.9%) were made at district or central hospitals, while 8.7% and 3.4% were made at private clinics and health centres respectively. Table 2 summarises the common types of cancer by gender, age and HIV status registered at NdiMoyo Palliative Care Centre from 2006 to 2013.

**Table 2:** Common types of cancer by gender, age and HIV status: NdiMoyo Palliative Care Centre, Malawi: 2006-2013

	Total		Male		Female		Children age <15 years		Adults age 15-59 years		Elderly age ≥60 years		HIV negative		HIV positive		HIV status unknown	
	n	%	n	%	n	%	n	%	n	%	n	%	n	%	n	%	n	%
Kaposi sarcoma	404	48.0	279	67.4	125	29.2	4	26.7	390	59.2	10	6.0	4	1.7	396	83.4	1	0.7
Cervix	178	21.1	-	-	178	41.6	0	0	122	18.5	56	33.3	83	35.6	51	10.7	44	32.8
Oesophagus	125	14.8	81	19.6	44	10.3	0	0	71	10.8	54	32.1	71	30.5	7	1.5	47	35.1
Liver	26	3.1	16	3.9	10	2.3	1	6.7	20	3.0	5	3.0	13	5.6	3	0.6	10	7.5
Breast	21	2.5	0	0	21	4.9	0	0	13	2.0	8	4.8	14	6.0	2	0.4	5	3.7
Skin	10	1.2	2	0.5	8	1.9	1	6.7	3	0.5	6	3.6	3	1.3	1	0.2	6	4.5
Bone	9	1.1	4	1.0	5	1.2	0	0	6	0.9	3	1.8	7	3.0	0	0	2	1.5
Burkett's lymphoma	8	1.0	4	1.0	4	0.9	8	53.3	0	0	0	0	4	1.7	1	0.2	3	2.2
Urinary bladder	7	0.8	2	0.5	5	1.2	0	0	6	0.9	1	0.6	6	2.6	0	0	1	0.7
Gastric	6	0.7	3	0.7	3	0.7	0	0	2	0.3	4	2.4	4	1.7	0	0	2	1.5
Eye	6	0.7	2	0.5	4	0.9	0	0	3	0.5	3	1.8	2	0.9	3	0.6	1	0.7
Others	42	5.0	21	5.1	21	4.9	1	6.7	23	3.5	18	10.7	22	9.4	11	2.3	12	9.0
Total	842	100	414	100	428	100	15	100	659	100	168	100	233	100	475	100	134	100

N= number of cancer cases in the group,%= percentage

### Palliative care services available at NdiMoyo Palliative Care Centre

NdiMoyo Palliative Care Centre was providing comprehensive holistic care in the form of consultations, counselling, hospitality and medication to address physical, psychosocial and spiritual needs of cancer patients. The services were provided at the centre and through outreach visits, home visits or road-side care (for homes that could not be accessed by vehicle) and annual patients’ party. Social support was provided to patients from poor families by providing them with food supplies and paying of school fees and uniforms for their children. For patients that died, bereavement visits and annual Remembrance Day were conducted with the deceased families. In terms of medication, pain was the most common symptom (99%) at the point of enrollment which was managed according to World Health Organisation analgesic ladder from paracetemol, asprin and NSAID on the first step to strong opioids on step 3. Oral liquid morphine was the most commonly used opioid. Vincristine was offered to all 404 KS patients and concurrently with HIV anti-retroviral therapy (ART) to 98.0% of KS patients. The common presentation of KS was swelling, pain, numbness and heat sensation on the affected site. For cervical cancer; vaginal bleeding and abnormal vaginal discharge were the common symptoms where Tranexamic^®^ acid for bleeding and antibiotics like metronidazole were prescribed. Dysphasia and odynophagia were the most common symptoms for esophageal cancer and dexamethasone and antifungals were prescribed. Other commonly used medications were amitriptyline, bisacodyl (along with morphine to prevent constipation) and nystatin. Patients were supplemented with natural medicine and nutrients in accordance with anamed Malawi guidelines. NdiMoyo had a large garden of natural medicines including Artemisia annua.

### Cancer survival rates among patients registered at NdiMoyo Palliative Care Centre 2006-2013

Cancer survival rates were analysed from a total of 830 patients with outcome data. Of the 830 patients; 65.2%, 49.2%, 39.4% were alive at 3, 6 and 9 months respectively. Only 31.8%, 18.0%, 12.5%, 7.8% and 6.0% were alive at 1, 2, 3, 4 and 5 years or more respectively. The overall median survival time for all cancers was 8.9 months. The survival rates for top five cancers at 1, 2, 3, and 4 years or more were; KS (n= 397): 47.1%, 30.2%, 21.4% and 13.1%; cancer of the cervix (n = 174): 31.0%, 10.3%, 5.2% and 2.9%; cancer of the oesophagus (n = 124): 4.0%, 2.4%, 1.6% and 1.6%; liver cancer (n = 26): 19.2%, 3.8%, 3.8% and 3.8%; breast cancer (n = 21): 9.5%, 0%, 0%, 0% respectively (Table 3, Table 4). Cox proportional hazard revealed that the hazard of death was 1.56 times higher in the age group of 50 years or more compared to those aged less than 50 years (p = 0.0001, Log Rank = 24.8, DF = 1). The hazard of death was 8.0% higher in females than in males although the results were not statistically significant (p = 0.05, Log Rank =3.75). There was no significant difference in the survival by marital status (married vs unmarried, p = 0.67), occupation (employed vs non-employed, p = 0.45) and education (educated vs non-educated p = 0.81). Females had 2 times increased hazard of death in Burkitts lymphoma, bone and urinary bladder cancer although the findings were not significant (p values of 0.340, 0.312 and 0.475 respectively). The results were not statistically significant most likely because the sample sizes for Burkitts lymphoma, bone and bladder cancer were very small; 8, 9, and 7 cases respectively. The hazard of death was found to be 1.2 times higher in females with esophageal cancer compared to males (p = 0.62, n = 125). Females with skin cancer were also found to have a higher hazard of death and the results were statistically significant (P = 0.001). The hazard of death was 36.5% higher in males with liver cancer compared to females, but the results were not statistically significant (p = 0.33) because of small sample size (n = 26). Survival in males and females for Kaposi's sarcoma was similar (hazard ratio = 1.0). Stratifying for age, there was an increased hazard of death in those aged 50 years or more for cervical, esophageal, bone and bladder cancers while for Kaposi's sarcoma, liver, breast, and skin cancers, the risk of death was higher in those aged less than 50 years.

**Table 3:** Cancer survival rates (%) by month: NdiMoyo Palliative Care Centre, Malawi: 2006-2013

	n	3	6	9	12	15	18	21	24	27	30	33	36	39	42	45	≥48 months
All cancers	830	65.2	49.2	39.4	31.8	26.7	22.4	20.1	18.0	16.3	15.4	14.6	12.5	10.8	9.9	9.0	7.8
Kaposi sarcoma	397	78.1	65.7	54.9	47.1	40.1	34.8	33.0	30.2	28.2	27.0	25.4	21.4	17.9	16.4	14.9	13.1
Cervix	174	74.1	54.6	42.5	31.0	25.3	19.0	14.4	10.3	7.5	6.3	5.7	5.2	5.2	4.6	4.0	2.9
Oesophagus	124	22.6	8.1	4.8	4.0	3.2	2.4	2.4	2.4	1.6	1.6	1.6	1.6	1.6	1.6	1.6	1.6
Liver	26	30.8	19.2	19.2	19.2	7.7	7.7	3.8	3.8	3.8	3.8	3.8	3.8	3.8	3.8	3.8	3.8
Breast	21	81.0	42.9	28.6	9.5	9.5	9.5	0	0	0	0	0	0	0	0	0	0
Skin	10	70.0	20.0	10.0	10.0	10.0	10.0	10.0	10.0	10.0	10.0	10.0	10.0	10.0	0	0	0
Bone	9	66.7	33.3	22.2	22.2	22.2	22.2	22.2	22.2	22.2	22.2	22.2	22.2	22.2	22.2	22.2	22.2
Burkett's lymphoma	8	62.5	50.0	25.0	25.0	25.0	25.0	25.0	25.0	25.0	25.0	25.0	25.0	25.0	25.0	25.0	12.5
Urinary bladder	7	57.1	42.9	14.3	0	0	0	0	0	0	0	0	0	0	0	0	0
Gastric	6	50.0	33.3	16.7	16.7	16.7	0	0	0	0	0	0	0	0	0	0	0
Eye	6	83.3	66.7	50.0	33.3	33.3	33.3	16.7	16.7	16.7	16.7	16.7	16.7	16.7	16.7	16.7	16.7

N= number of cancer cases in the group,%= percentage

**Table 4:** Median cancer survival rates: NdiMoyo Palliative Centre, Malawi: 2006-2013

	n	Median survival time (months)	95%CI
All cancers	830	8.9	-
Kaposi's Sarcoma	497	23.9	14.2-33.6
Cervix	174	9.6	7.4-11.7
Oesophagus	124	1.6	1.1-2.12
Liver	26	1.0	0.5-1.6
Breast	21	5.6	4.9-6.3
Skin	10	4.9	2.0-7.8
Bone	9	3.7	0.9-8.3
Burkett's lymphoma	8	4.8	0.3-9.3
Urinary bladder	7	6.6	5.2-7.1

N= number of cancer cases in the group, CI= Confidence interval

## Discussion

Data on cancer survival from eastern and southern Africa are scarce because of lack of functional population-based registries that collect data regularly [14]. This paper has provided insight on common types of cancers and cancer survival from the time of diagnosis in Malawi using the available seven- year follow up data of over 800 patients from a palliative care centre. The pattern on common types of cancer by gender and age was similar and comparable to national population-based cancer registry estimates of 2010. Consistent with population-based cancer registry data; cancer of the cervix (41.6% vs 45.4%), Kaposi′s sarcoma (29.2% vs 21.1%), cancer of the oesophagus (10.3% vs 8.2%), breast cancer (4.5% vs 4.6%) were the most frequent in females. In males; Kaposi′s sarcoma (67.4% vs 50.7%), cancer of the oesophagus (19.6% vs 16.9%), and cancer of the liver (3.9% vs 1.5%) were most common in males [14]. This could suggest that estimates on common types of cancer by gender and age from palliative care centre could be used a proxy where population-based cancer registry is un-functional or not regularly conducted and/ or in triangulation provided that sample size is large enough.

This study also highlighted that cancer patients in Malawi were young with mean age of 33 years and only one third (27.6%) were aged 60 years or more. More than half (56.4%) of cancer patients were HIV positive. Similar findings were also reported by other studies [11, 14–16]. Sustained efforts on high universal coverage for HIV and malaria prevention and treatment and introduction of human papilloma virus vaccine could reduce the cancer burden of Kaposi′s sarcoma, Burkitts lymphoma and cervical cancer respectively [17].

This study, for the first time in Malawi, has provided comprehensive insight on cancer survival. Survival from the time of diagnosis was very low with a median survival time of 9 months, range 1 month for liver cancer to 24 months for Kaposi′s sarcoma patients. Only 6% of the patients survived for 5 years or more, range 0% for breast, skin, urinary bladder and gastric cancer to 13.1% in patients with Kaposi′s sarcoma. Studies from other countries in east and southern Africa (Zimbabwe, Uganda and Kenya) have also reported very poor cancer survival estimates [7–9]. Inadequate/lack of cancer screening programmes, late presentation of patients, late diagnosis, inadequate/lack of diagnostic facilities, unavailability of treatment, limited access of patients to diagnosis and treatment sites and HIV have been documented as some of factors that are contributing to poor cancer survival in this region [7–10]. All these factors are present in Malawi and therefore addressing them may lead to early detection, diagnosis and treatment which in turn will lead to improved cancer survival [14, 16]. Some of the efforts underway in Malawi to improve early detection, diagnosis and treatment of cervical cancer were the national cervical cancer screening programme using visual inspection with acetic acid (VIA) which started in 2004. By the end of 2013, there were over 100, 50 and 3 sites providing cervical cancer screening, cryotherapy and loop electrical-surgical excision procedure (LEEP) respectively. Over 20,000 women were screened in 2013. Of these, 7.1% were VIA positive and 6.3% suspected cervical cancer and 8.3% had other gynaecological conditions, the commonest being cervicitis [18]. Promotion of community awareness on common cancers, scaling up of histopathology laboratory services and establishment of radiotherapy services may improve early detection, diagnosis and treatment thereby improving cancer survival in Malawi.

### Limitations of the study

This study was facility-based and only from one palliative care centre hence the survival estimates presented could not be generalised. Population-based cancer survival estimates from cancer registry could have been the best but data collection by the Malawi Cancer Registry is irregular, mainly confined to one district (Blantyre) and does not have follow-up data [14]. The other limitation was that sample sizes for some cancers were small making survival estimates for those specific cancers less reliable. Lack of information on the grade or stage of cancer was another limitation although this was not specific to this study but a general problem in Malawi where majority (>80%) of cancer cases are not laboratory confirmed and clinical staging is also not usually done [14]. Nonetheless, this study has provided an insight on cancer survival estimates based on the available seven year follow-up data of over 800 cancer patients which could be used inform interventions and strategies to improve early detection, diagnosis, treatment and palliative care services in Malawi.

## Conclusion

In Malawi, cancer survival from the time of diagnosis was very poor with median survival time of about 9 months and only 6% of patients survived for 5 years or more. Improvement of public awareness and early detection, diagnosis, treatment options including chemotherapy and radiotherapy, and palliative care services could improve cancer survival.
